# Comparative of fibroblast and osteoblast cells adhesion on surface modified nanofibrous substrates based on polycaprolactone

**DOI:** 10.1007/s40204-016-0059-1

**Published:** 2016-12-08

**Authors:** Fereshteh Sharifi, Shiva Irani, Mojgan Zandi, Masoud Soleimani, Seyed Mohammad Atyabi

**Affiliations:** 1 Department of Biology, School of Basic Sciences, Science and Research Branch, Islamic Azad University, Tehran, Iran; 2 Department of Biomaterials, Iran Polymer and Petrochemical Institute, Tehran, Iran; 3 Department of Hematology, Faculty of Medical Science, Tarbiat Moddares University, Tehran, Iran; 4 Department of Pilot Biotechnology, Pasteur Institute of Iran, Tehran, Iran

**Keywords:** Plasma treatment, Nano-topography, Electrospinning, Human fibroblast cells (HDFs), Osteoblast cells (OSTs)

## Abstract

One of the determinant factors for successful bioengineering is to achieve appropriate nano-topography and three-dimensional substrate. In this research, polycaprolactone (PCL) nano-fibrous mat with different roughness modified with O_2_ plasma was fabricated via electrospinning. The purpose of this study was to evaluate the effect of plasma modification along with surface nano-topography of mats on the quality of human fibroblast (HDFs) and osteoblast cells (OSTs)-substrate interaction. Surface properties were studied using scanning electron microscopy (SEM), atomic force microscopy (AFM), contact angle, Fourier-transformation infrared spectroscopy. We evaluated mechanical properties of fabricated mats by tensile test. The viability and proliferation of HDFs and OSTs on the substrates were followed by 3-[4, 5-dimethylthiazol-2-yl]-2, 5-diphenyltetrazolium bromide (MTT). Mineralization of the substrate was determined by alizarin red staining method and calcium content of OSTs was determined by calcium content kit. Cells morphology was studied by SEM analysis. The results revealed that the plasma-treated electrospun nano-fibrous substrate with higher roughness was an excellent designed substrate. A bioactive topography for stimulating proliferation of HDFs and OSTs is to accelerate the latter’s differentiation time. Therefore, the PCL substrate with high density and major nano-topography were considered as a bio-functional and elegant bio-substrate for tissue regeneration applications.

## Introduction

In tissue bioengineering, scientists have attempted to simulate the microenvironment and surface topography of the substrates for improving and accelerating the cellular responses, including attachment, migration, proliferation, and expression of differentiated phenotypes (James et al. 2011; Besunder et al. 2010). Tissue engineering is a new promising field for biomedical engineering to facilitate and maintain, improve, regenerate and replace the functional living tissues (López-Pérez et al. 2010; Irani et al. 2013). Acclaiming the potential of the three-dimensional (3D) substrates is to enhance tissue regeneration as an important subject in the field of tissue engineering (Irani et al. 2013; Farooque et al. 2012). Indeed, one of the major challenges in bioengineering of tissue is to design and fabricate the substrate that can promote to restore functionality of tissue (Farooque et al. 2012; Moon et al. 2012). Electrospun nano-fiber substrates have many favorable properties, such as high surface area-to-volume ratio, nano-topographical features, and polymer concentration (James et al. 2008; Kumbar et al. 2008). Furthermore, electrospinning accompanying nanotechnology process emerges as a versatile and particular technique to form fibers with diameter in the range from µm down to nm to produce fiber substrates that simulate the architecture of native extracellular matrix (ECM) (Irani et al. 2013; James et al. 2008; Kluger et al. 2010). Thus, nano-fibers were fabricated through electrospinning technique due to the advantages to repair and regenerate the tissue in bioengineering (Cheng et al. 2014; Guadalupe et al. 2015). Materials as substrates with physical, mechanical, and biological properties to simulate target tissue are keys in designing the proper microenvironment to achieve the best cell responses (Farooque et al. 2012). Biomaterials used in fabrication of the substrates are commonly based on natural polymers (such as: collagen, chitosan and gelatin) and synthetic polymers (such as: poly (lactic acid) (PLA), poly (ɛ-caprolactone) (PCL), polyglycolic acid (PGA) (Irani et al. 2013). Poly (ɛ-caprolactone) (PCL) is one of the favorable synthetic biomaterials, because it has many extensive properties like: biocompatibility, biodegradability, mechanical strength and flexibility (James et al. 2008; Lee et al. 2008). Although bio-substrate provides desired microenvironments for cell seeding, but hydrophobic nature of PCL gives rise to low cell loading in the initiatory step of the cell culture leading to less cellular adhesion, migration, and proliferation (Besunder et al. 2010; Lee et al. 2008). Plasma treatment results in physical and chemical changes by introducing new chemical functional groups, improvement in hydrophilicity and surface permeability (Besunder et al. 2010; Lee et al. 2008; Mirzadeh et al. 2015). To improve the biocompatibility, PCL substrate with different roughness was selected (Chan et al. 2008) and treated with O_2_ plasma technique. Pure oxygen gas ignited in a plasma chamber induces the bombardment of a homogeneous compound of charged particles, such as electrons, ions, excited molecules, and neutral radicals of UV radiation on the polymer surface (Besunder et al. 2010). Hence, the purpose of this study was to investigate the effect of surface modification of electrospun PCL substrate on cell behavior in comparison to the unmodified surface substrates, such as HDFs and OSTs.

## Methods

### Materials

The human fibroblast cells and osteoblast cells were obtained from the Cell Bank Stem Cell Technology Institute, Iran. PCL, was purchased from Sigma Aldrich (average molecular weight of 80,000 Da), Germany. 3-[4,5-dimethylthiazol-2-yl]-2,5 diphenyltetrazolium bromide MTT, alizarin red staining, dexamethasone and ascorbic acid (Sigma) were purchased from Sigma, Germany, UK, Roswell Park Memorial Institute-1640. The calcium content assay kit was purchased from Parsazmun, Iran. Dulbecco’s Modified Eagle’s Medium (DMEM), Fetal bovine serum, and trypsin 0.25%/EDTA solution was obtained from Gibco, Canada. Home-lab phosphate-buffered saline (PBS) reagents (NaCl, KCl, Na_2_HPO_4_, and KH_2_PO_4_) were prepared and used. The test groups for this study were as follows: (1) PCL nano-fiber different morphologies modified by plasma; (2) unmodified PCL nano-fiber with different morphologies. In continuation, the morphology of the nano-fiber, cell viability, morphology of the cells on the nano-fiber and related results are presented.

### Nano-fibrous PCL substrate fabrication

Nano-fiber PCL substrates were made by electrospinning technique (CO881007NYI, Asian Nanostructures Technology Co), Iran. PCL (13 wt%) was dissolved in acetic/formic acid for a day under stirring conditions. The polymer solution was loaded into a 5-ml plastic syringe fitted with a needle with a tip diameter of 0.4 mm. Polymer solutions were fed into the needle tip using a syringe at a flow rate of 0.9 ml/h; in a drum collector (50 mm wide and 70 mm thick) covered with aluminum sheet. The distance between the needle tip and the aluminum sheet was maintained at 160 mm. By applying a high different voltage (15–25 kV), the polymer solution formed a Taylor cone at the needle tip and a positively charged jet was stretched toward the collector. PCL nano-fiber substrates were collected by 10, 40 and 70 min electrospinning prolonged collecting time causes formation of different surface texture which leads to different surface morphology, roughness and waviness.

### Characteristics of the surface modified PCL nano-fiber substrates

#### Scanning electron microscopy (SEM) and atomic force microscopy (AFM)

**Fig. 1:**
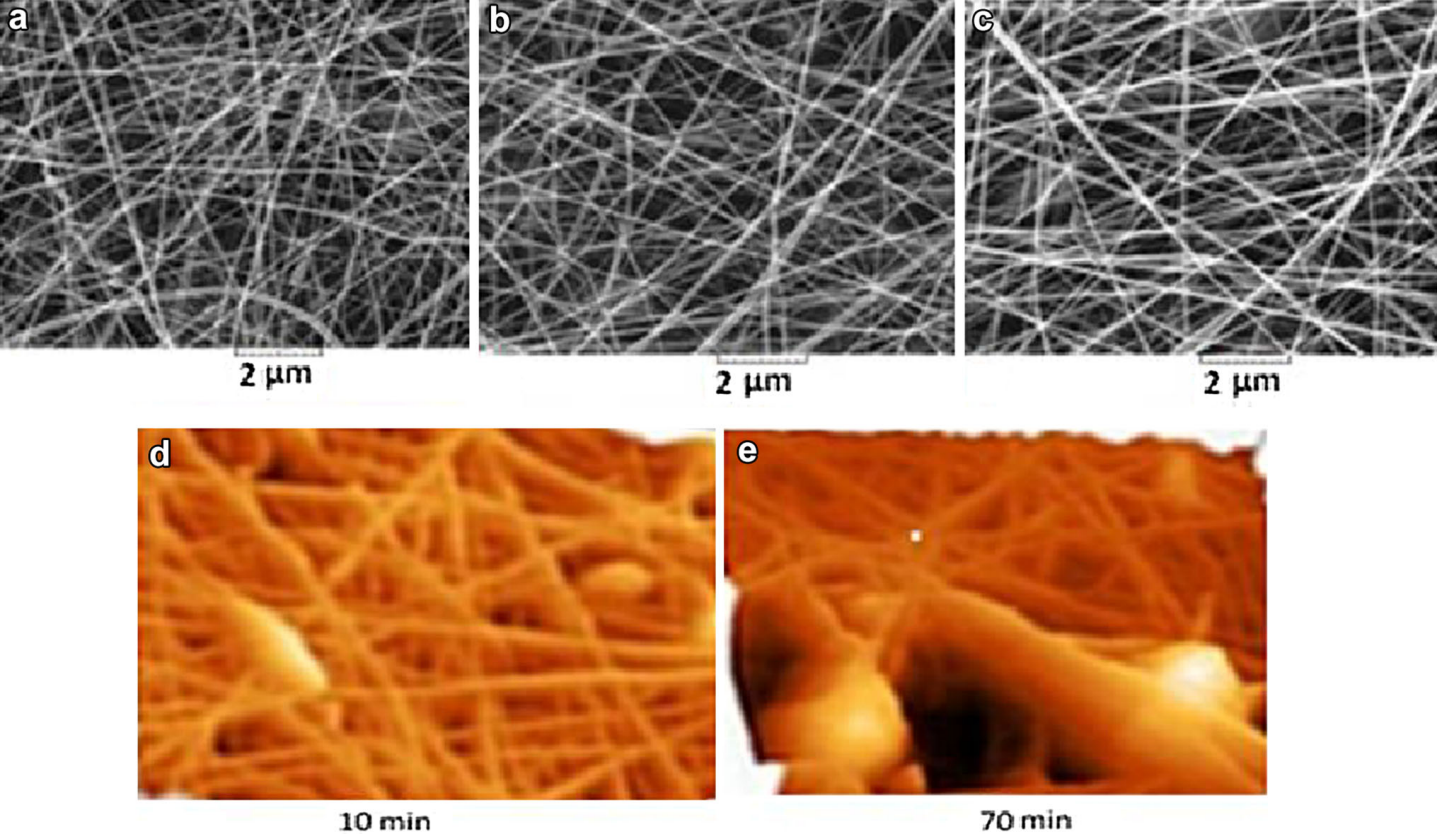
Comparison of the morphological structure of three PCL substrates using SEM images and atomic force microscopy (AFM) phase images of the PCL nano-fiber substrate. **a** The nano-fiber PCL substrate prepared by electrospinning at 10 min collecting time; **b** 40 min collecting time; **c** 70 min collecting time (*scale bar* 2 µm); **d** the PCL nano-fiber substrate collected at 10 min and **e** the PCL nano-fibrous substrate collected at 70 min

Electrospun nano-fiber substrates were coated with gold and observed under scanning electron microscope (VEGA/TESCAN) at an accelerating voltage of 20 kV. Before seeding the cells on the surface of the substrates, the surface morphology and roughness of the PCL nano-fiber substrates were collected by 10, 40 and 70 min electrospinning and analyzed by atomic force microscopy (AFM: model Dual Scope C26 of DME Co.). The AFM images were recorded in tapping mode and the surface roughness [the root mean square (Sq)] for the samples with different collecting times, was calculated (Fig. 1). The horizontal and vertical lengths of the cross-section were measured by cross-section analysis technique.

#### Plasma surface modification of electrospun nano-fibers

O_2_ plasma treatment of electrospun PCL nano-fiber substrates were carried out using Nano-diener plasma cleaner (Stem Cell Technology Institute, Electronic diener, Iran). Nano-fibers were placed in a chamber of the plasma cleaner and subjected under plasma discharge for 5 min with low frequency power set as 30 W under vacuum mode.

#### Surface chemical characterization and assessment of surface hydrophilicity

The composition of the surface plasma-treated PCL nano-fiber substrates was evaluated by an Equinox ATR-FTIR (55, Bruker, Germany). The spectra were recorded from 400 to 4000 cm^−1^ with a 4 cm^−1^ resolution. The degree of hydrophilicity for PCL nano-fiber substrates was measured by contact angle before and after O_2_ plasma treatment in 5 μl of deionized water mounted on the surface of substrates. All measurements were conducted at room temperature.

#### Mechanical property test

A Tensile System model STM-20 (Iran) equipped with a 4.19 N load-cell was used to evaluate tensile strength (TS), elongation-at-break (EAB) of nano-fiber substrate before and after plasma surface treatment according to the ASTM standard method D882. Substrate strips of 10 × 60 mm were measured while initial grip separation and mechanical crosshead speed was set at 30 mm and 10 mm/min, respectively. Each type of substrate was analyzed by at least five replicates.

### Cell-substrate interaction study

HDFs and OSTs were cultured in the Dulbecco’s modified Eagle’s medium (DMEM) (Gibco, Canada) containing 10% fetal bovine serum (FBS) (Gibco, Germany). Upon confluence (rare), HDFs and OSTs were trypsinized and resuspended in the medium at a concentration of HDFs (10^3^ cells in 30 µl medium on 0.5 × 0.5 cm substrates) and OSTs (3 × 10^4^ cells in 30 µl medium on 0.5 × 0.5 cm substrates) which were chosen to give the maximal response without restricting their spread ability. The bottoms of 24-well cell culture plates were totally covered with substrates. The cell suspension was also divided at the bottom of the empty well for positive control. HDFs and OSTs were seeded on substrates. After 2 h of incubation, 1 ml fresh medium was added onto each well. The cell-substrate constructs were maintained in an incubator (37 °C and 5% CO_2_) for further examination. One day after OSTs were transferred on the substrates for promoting the osteoblast phenotype, the medium was supplemented with 600 µg ml^−1^ dexamethasone and 5 mg ml^−1^ ascorbic acid. The medium of each well was changed twice per week. After the cell-seeded substrates were rinsed twice with PBS (pH 7.4) and immersed in PBS containing 4.5% glutaraldehyde for 3 h to fix the cells, they were dehydrated through a graded series of ethanol (from 60 to 100%) and vacuumed dried overnight. The dry cell-seeded substrates were mounted on a sample holder and their morphology was characterized by a Vega scanning electron microscope (TESCAN) to study the attached cells and evaluate their proliferation.

### MTT assay

The viability and proliferation of the HDFs and OSTs were also measured using 3-[4,5-dimethylthiazol-2-yl]-2,5-diphenyltetrazolium bromide (MTT); cell viability of HDFs tested at 24, 48, 72 h, 14 and 28 days and the OSTs tested at 24, 48, 72 h, 7, 10, 14 and 18 days from MTT assay. First, a batch of 100 μl MTT solution with 900 μl medium was incubated with the cells on the PCL substrate in the wells at 37 °C for 3 h (*n* = 3 for each measurement day). After 3 h, dimethyl sulfoxide solution (DMSO, Sigma Chemical, Germany) was added to dissolve formazan crystals. The absorbance values of formazan solutions, obtained from the above given substrates, were measured using an ELIZA reader at 570 nm (Bio-Tek ELx 800). All calculations were made using SPSS statistical software.

### Alizarin red staining

The OSTs were cultured on substrates (3 × 10^4^ cells/each substrate) and incubated for 4, 8, 10 days. The substrates were washed with PBS at pH 7.4, and fixed with 10% paraformaldehyde solution in PBS (pH 7.4) for 30 min. In the next step, alizarin red stain (10 mg/ml) was added and incubated for 45 min under ambient condition. At the end, stained substrates were observed with an inverted microscope (Bell, INV-100FL).

### The calcium content assay

The calcium minerals sediment on OSTs is quantified. Therefore, this marker was quantified through cresolphthalein complexone method with a calcium content assay kit (Parsazmun, Tehran, Iran). OSTs were cultured on nano-fiber substrates (3 × 10^4^ cells/each substrate) for 72 h, 7, 10 and 14 days. Extraction content of calcium from OSTs on the substrates was performed by 0.6 N HCL (Merck, Darmstadt, Germany). The reagent was added into calcium solution and the absorbance was measured at 570 nm by an ELISA plate reader (Bio-Tek ELx800).

### Statistical analysis

Data were obtained in triplicate, averaged, and expressed as mean ± standard deviation (SD). Statistical analysis was carried out using one way analysis of variance (ANOVA). A value of *p* ≤ 0.05 was considered statistically significant. Data are recorded in triplicate.

## Results

### Fiber morphology of PCL nano-fiber substrates

The surface morphology of the electrospinning nano-fibers is shown in Fig. 1a–c. The SEM micrographs of nano-fibrous PCL substrate prepared by electrospinning at (a) 10 min collecting time; (b) 40 min collecting time; (c) 70 min collecting time.

SEM results illustrated the fiber density differences in nano-fibers with increasing the collecting time from 10, 40 and 70 min (Fig. 1). Referring to the Fig. 1, it can also be said that as the collecting time increases the Sq also increases. On the other hand, the alteration in the PCL nano-fibers surface topography through longer collecting time has been imaged by AFM, and the root mean square of the height distribution (Sq) for the samples with different collecting times was calculated and Sq is 205, 225, and 243 nm for substrate collected at 10, 40 and 70 min, respectively. AFM images are shown in Fig. 1d, e, (d) presented the PCL nano-fibrous substrate collected at 10 min and (e) illustrated the PCL nano-fibrous substrate collected at 70 min.

### Determination of surface chemistry and contact angle of PCL nano-fibers

**Fig. 2:**
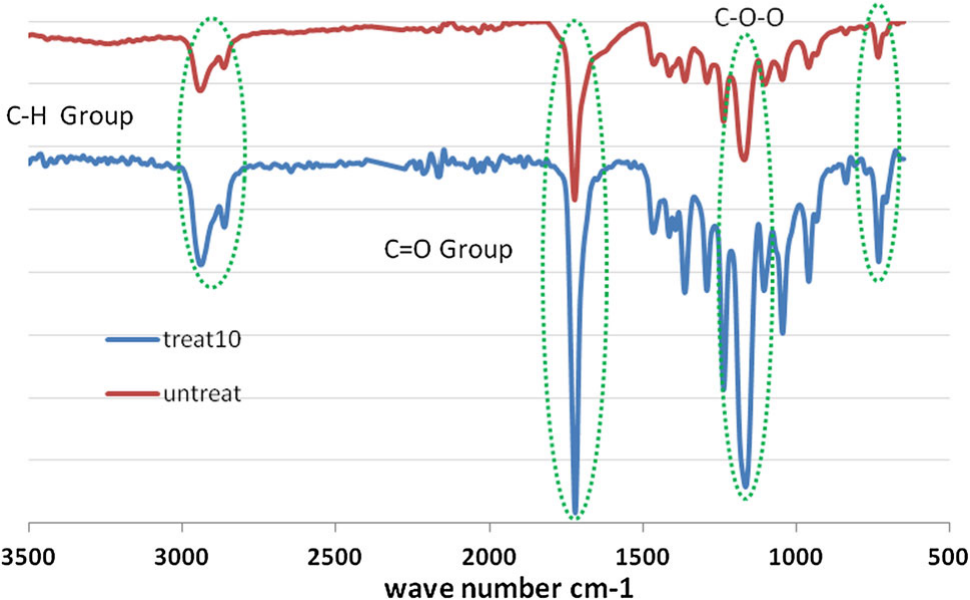
FTIR absorbance spectra of the oxygen plasma-treated PCL nano-fiber and pristine substrates prepared by electrospinning at 70 min collecting time

The surface chemistry of pristine and treated PCL nano-fibrous substrates was investigated by FTIR to confirm the shifts in absorption band of active groups created by oxygen plasma modification (Fig. 2). The data from contact angle measurement, before and after surface modification, are listed in Table 1. The results demonstrate that the surface modification by O_2_ plasma can improve the hydrophilic properties of the fabricated substrates.

**Table 1:** Contact angles of oxygen plasma modified nano-fibers collected at 70 min

Property	Treated PCL	Untreated PCL
Contact angle	0°	122 ± 5°

### Mechanical property test

The tensile character of PCL nano-fibers, modified and unmodified by O_2_ plasma, was evaluated and tensile strength as well as elongation-at-break are listed in Table 2. It seems that there is no significant difference between modified and unmodified substrates statistically and both groups show almost the same tensile strength and elongation-at-break. It can be concluded that the plasma surface modification of substrate did not cause a distinct change in the mechanical properties of the samples, while the biological property of the substrates has been improved. Therefore, the results show that without loss of mechanical properties, improvement in cell behavior has been observed.

**Table 2:** Mechanical properties of the fabricated nano-fiber substrate before and after plasma surface modification

Sample	Tensile strength (MPa)	Elongation-at-break (%)
Untreated PCL	2.0 ± 0.4	47.4 ± 5.5
Plasma-treated PCL	1.8 ± 0.3	53.6 ± 4.7

### Cell proliferation

**Fig. 3:**
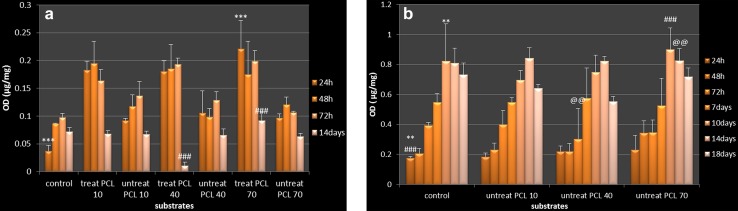
Cell viability versus culturing time: **a** cell viability of human fibroblast cells (HDFs) tested at 24, 48, 72 h, 14 and 28 days from MTT assay; **b** cell viability of the osteoblast fibroblast cells (OSTs) tested at 24, 48, 72 h, 7, 10, 14 and 18 days from MTT assay. Data are expressed as mean ± SD (*n* = 3). Data are subject to Dun-net one way analysis of variance (ANOVA), ***p < 0.001. (Treat PCL 10: O_2_ plasma-treated PCL 10 min collecting time, treat PCL 40: O_2_ plasma-treated PCL 40 min collecting time, treat PCL 70: O_2_ plasma-treated PCL 70 min collecting time, untreat PCL 10: untreat PCL 10 min collecting, untreat PCL 40: untreat PCL 40 min collecting, untreat PCL 70: untreat PCL 70 min collecting)

Cellular attachment and proliferation of HDFs on PCL substrate, modified with O_2_ plasma, showed higher rate compared to the unmodified and positive control in the first day. In fact, these results confirmed that PCL nano-fibrous substrates modified by O_2_ plasma with maximum rough (Fig. 3a).

For OSTs, the cell proliferation gradually increased on PCL nano-fibers from 1 to 10 days. Despite this, after 14 days from cell transfer onto the substrates there has been a downward trend in growth and proliferation (Fig. 3b). Based on the obtained results from MTT test the inter-relationship between the cell behavior and the substrate drastically depends on the density of nano-fibers and changes in surface nano-topography.

### Cell morphology on the nano-fibers

**Fig. 4:**
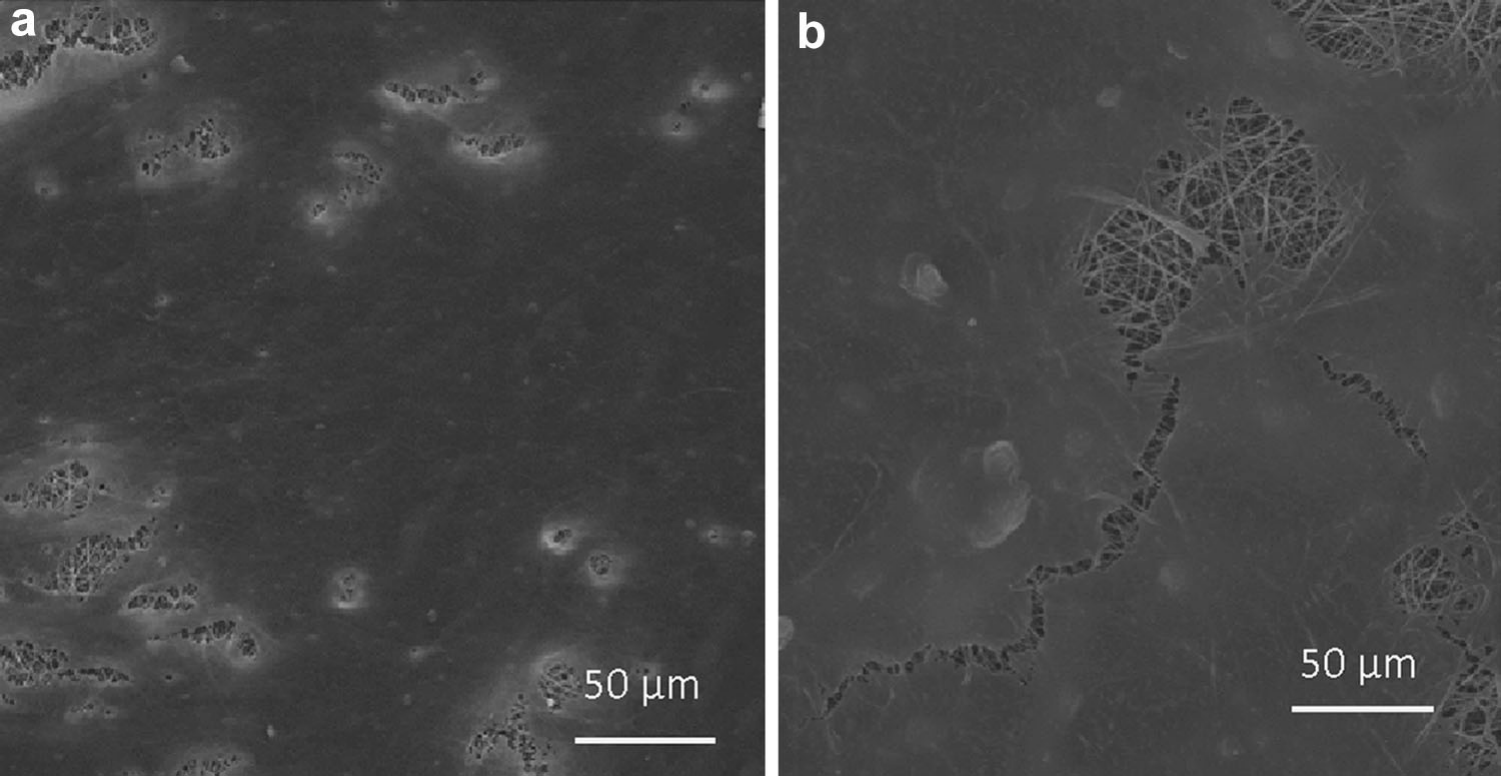
SEM photomicrographs of OSTs (*left*) and HDFs (*right*) electrospun substrate at 10 min collecting time, cultured for 48 h. The substrates are shown in *circles*

The morphology and distribution of HDFs and OSTs on electrospun PCL nano-fibers were examined by SEM (Fig. 4). Based on these results, it can be stated that fusiform morphology of HDFs and OSTs on PCL nano-fiber substrates simulates the microenvironment for cellular regeneration.

### Alizarin red staining

**Fig. 5:**
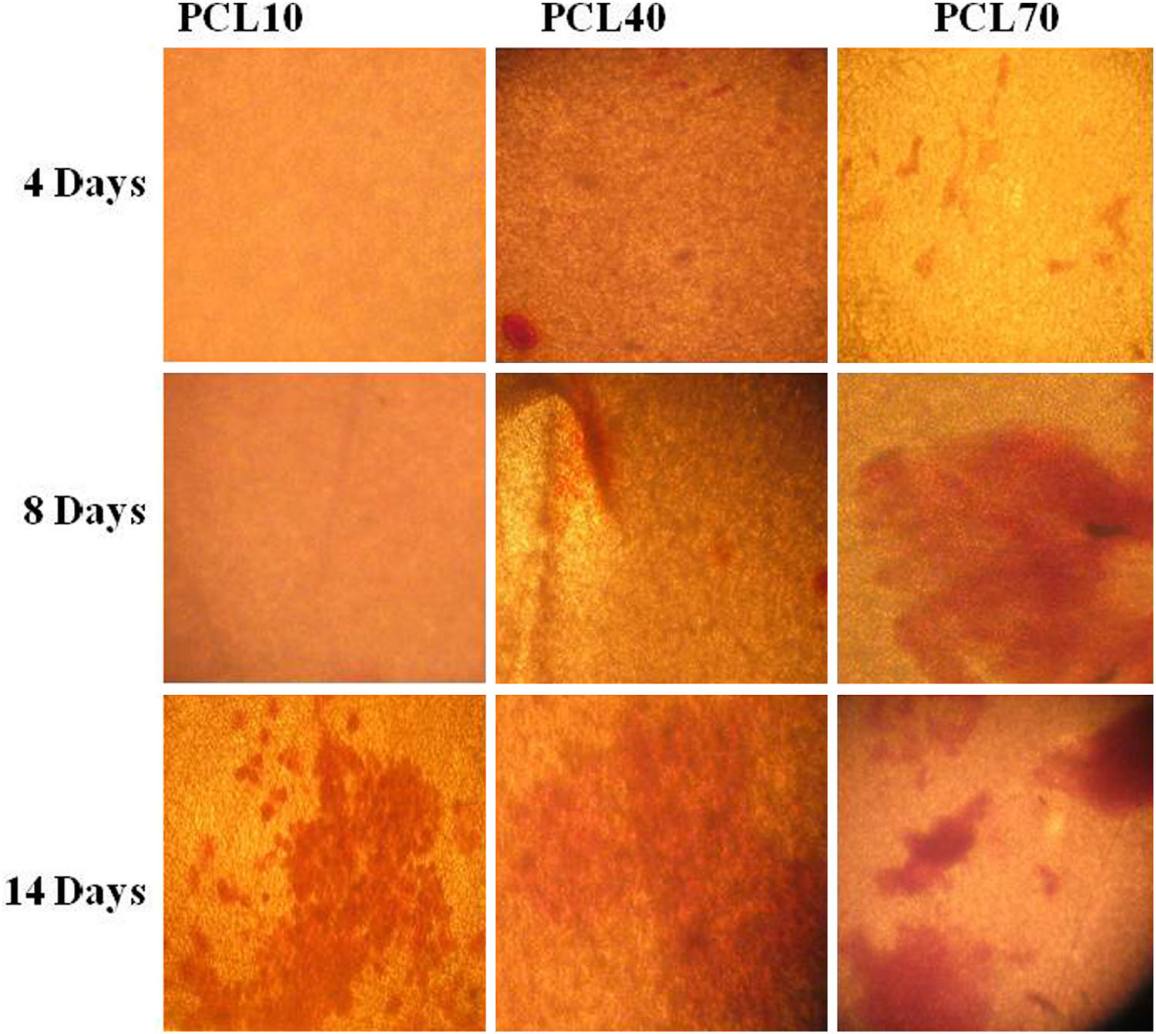
The micrographs of calcium staining (*alizarin red*) are shown after 4, 8 and 14 days culture under osteogenic induction medium, *scale bars* (100 μm)

The results of this test are shown in Fig. 5. The substrates designed as well as the osteogenic supplements displayed inducer proliferation and differentiation process. During the experiment substrate with high density, collected at 70 min, led to increased calcification of their matrix.

### The calcium content assay

**Fig. 6:**
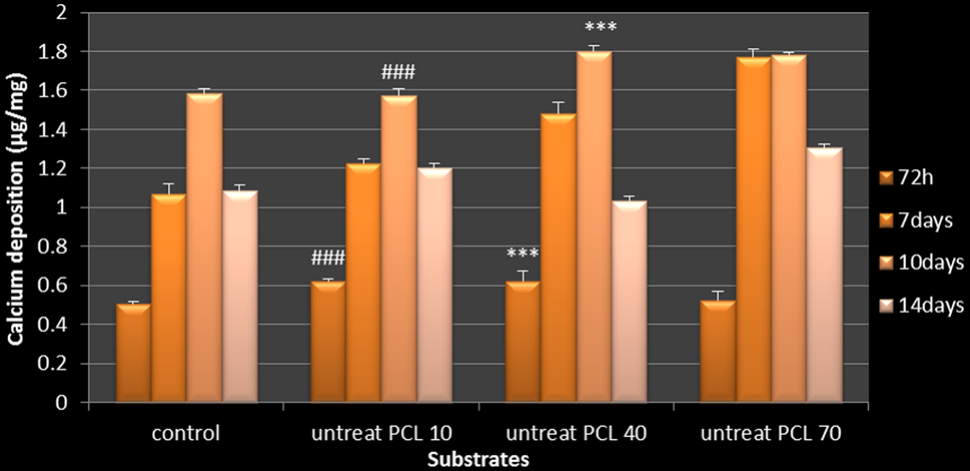
Calcium content of osteoblast cells during osteogenic differentiation, *asterisk* shows significant difference between the substrates on each day at p*** < 0.001. (Untreat PCL 10: untreat PCL 10 min collecting, untreat PCL 40: untreat PCL 40 min collecting, untreat PCL 70: untreat PCL 70 min collecting)

The calcium content of OSTs on the substrate and control is shown in Fig. 6. The substrate with 70 min collecting time illustrated highest progressive calcium deposition at all the time points.

## Discussion

The aim of this study was to evaluate the effect of changes on surface topography of the PCL-based substrate collected at three different times. Topography of the produced substrates guides the cells toward functional living tissue. PCL-based nano-fibrous substrates are considered as convenient options for being used as microenvironment in tissue engineering (Hui et al. 2010; Dhanasekaran et al. 2014).

### Fabrication of nano-fibers

Design and fabrication of substrates to provide a suitable platform optimized for producing and secretion of ECM by cells can lead to formation of a functional organ or tissue (Irani et al. 2014; Swindle-Reilly et al. 2014). According to the hypothesis in this study, changes in process parameters in electrospinning technique result in the preparation of nano-fibers with desirable and optimal characteristics. PCL-based nano-fibers in three different collecting times (10, 40, and 70 min) were prepared by electrospinning technique.

### Morphology study

To evaluate the morphology of the electrospun nano-fibers, micrographs were prepared by SEM. Differences in the density of the collected nano-fibers are clearly observed in Fig. 1, which also increases by increasing the spinning time. Recently, a great attention has been paid to the substrates with different surface roughness which is one of the new innovations to achieve better cellular behavior. One of the obstacles in the preparation of three-dimensional environment for tissue engineering is to provide a substrate in which the cells, as in nature, are accommodated and connected to each other and the surrounding matrix. In fact, surface treatment and surface roughness improvement of the substrates lead to better and faster interaction between the cells and matrix and facilitate cellular exchange of nutrients, waste products and respiratory gases. A variety of methods and materials are used to achieve surface treatment on substrates. Acevedo et al. used different doses of gamma radiation to sterilize the gelatin/chitosan/hyaluronan substrates. The use of gamma rays led to the creation of roughness on the surface of substrates. The results showed that a dosage of 25 kGy produced a rough microstructure with a reduction of the porosity (from 99 to 96%) which led to increased cell proliferation (compared with lower doses of gamma radiation) (Acevedo et al. 2013). Kumar et al. also used a solution containing 10% dichloromethane and 90% acetone to create the roughness on the PCL substrate. This result demonstrated the improvement of the cells’ behavior on the substrate roughness compared to the substrate without roughness (Farooque et al. 2012). In this study, electrospun PCL-based nano-fibers which have been produced by various time intervals of spinning provide different roughness (Mirzadeh et al. 2015). As it can be seen in Fig. 1, by increasing the spinning time, heterogenous density of nano-fibers was also increased. To show different roughness, the AFM was used. As it can be seen in these images, different spinning time created different roughness (Fig. 1).

### Effect of surface modification on surface hydrophilicity

Besides the PCL advantages in tissue engineering, inherent hydrophobicity of this polymer can result in low attachment of the cells in the first step of the cell transfer on the substrate, and consequently low rates of cell proliferation and growth (Besunder et al. 2010; Atyabi et al. 2016). Krishnan et al. used polyvinyl alcohol/xylene blend nano-fiber produced by electrospinning which is exposed to glutaraldehyde vapor at different time intervals. The results indicated that increasing the evaporation time led to an increase in crosslinking and also roughness of the substrates and enhanced the growth and proliferation of cells on the substrate (Krishnan et al. 2012). In studies conducted by Mavis et al. and Guarino et al. which led to improved connectivity and ossification, calcium triphosphate was used in PCL substrates for bone tissue engineering (Demirtas et al. 2009, Ambrosio and Guarino 2008). Various methods have been used to improve hydrophilicity of polymers which have led to the provision of active groups, such as (carboxyl, amino and hydroxyl) on the surface of the substrate. One of these methods is plasma surface modification treatment which helps to improve the biological properties of the polymers (Atyabi et al. 2016; Desai and Singh 2004; Hourston and Lane 1993). Plasma treatment plays a vital role in improving the surface hydrophilicity of the polymer. To modify the surface of PCL substrates prepared by electrospinning method, Prabhakaran et al. used air plasma and biological substrates, made of collagen and PCL, to investigate the cellular behavior. Indeed, the measurement of contact angel of modified and unmodified substrates indicates that the improvement of hydrophobicity is due to the increase of oxygen groups on the surface (Chan et al. 2008). Hence, plasma is a fast, comfortable, and low-cost method without the use of solvent, which can create active sites on the surface of the polymers (Gazicki and Yasuda 1982; Greene et al. 2003; Grace and Gerenser 2003). In this study, oxygen plasma was used for surface modification of electrospun nano-fibers. Induction of O_2_ plasma on PCL nano-fiber substrates led to significant improvement of moisture variability.

### Contact angle changes confirmed by FTIR spectroscopy

Before and after surface modification, contact angle was measured. The measurement of contact angle depends on hydrophilicity of nano-fiber electrospinning collected at three different times. Lower contact angle shows a high level of hydrophobicity of the substrates (Forch et al. 2009). Better cellular attachment may be related to the hydrophilicity which promotes the rate of cellular attachment (Irani et al. 2014). The measurement of contact angles of O_2_ plasma modified nano-fibers collected at 70 min is shown in Table 1. As it can be seen, contact angle for PCL modified nano-fiber substrate by O_2_ plasma is close to zero, which indicates the improved hydrophilic properties compared to unmodified PCL nano-fibrous substrate. The presence of polar functional groups (e.g., carbonyl and carboxyl) changes a hydrophobic surface to a hydrophilic surface (Besunder et al. 2010). To evaluate their chemical composition, samples were tested by ATR to confirm that chemical structural changes in nano-fibers have a significant impact on cell behavior. FTIR spectra of unmodified and modified nano-fiber substrates are presented in Fig. 2. As shown in Fig. 2, a weak peak is seen around wavenumber of 2852–2940 cm^−1^ on surface modified nano-fiber, especially on samples with longer collecting times that can be referred to symmetric and asymmetric CH_3_ and asymmetric CH_2_ groups. The existence of absorbance peaks at 1720 cm^−1^ for C=O functional groups and the absorbance peak at 1420 cm^−1^ for –COO functional groups, presented in Fig. 2, corresponds to changes on a modified nano-fiber substrate. Results obtained from FTIR method show the surface modification of PCL nano-fibrous substrate by the presence of hydrophilic functional groups.

### Mechanical test

Using plasma for surface modification of biomaterial has many advantages like improving hydrophilicity and incorporating chemical functional groups. Another advantage for this surface modification technique is that it does not affect the mechanical properties (Chan et al. 2008; Johnson et al.^2009^). This claim was confirmed by tensile test. Tensile property of PCL nano-fibers was measured before and after plasma surface modification. Finally, no significant difference between the modified and unmodified PCL nano-fibers was evaluated (Table 2).

### Cell attachment and proliferation study

HDFs and OSTs culturing were used to confirm that PCL substrates with different roughness have a great impact on cell behavior. Therefore, two groups of substrates were defined: (1) PCL nano-fibrous substrate modified with oxygen plasma for culturing of HDFs and (2) PCL unmodified nano-fibrous substrate for culturing OSTs and HDFs. The cells were cultured on the surface of nano-fibrous substrates in different time intervals, HDFs: 1, 2, 3, 14 days and OSTs: 1, 2, 3, 7, 10, 14 and 18 days. In this study, MTT assay was used on both defined nano-fibrous substrates to analyze HDFs and OSTs growth rate. With regards to HDFs behavior on the surface of modified nano-fibrous substrate, the initial cellular attachment on the first day was significantly more than positive control as well as the unmodified substrates. The result demonstrates the direct effect of O_2_ plasma on the surface to improve the basic cellular attachment. On the third day, modified substrates with 10 min collecting time show lower cell growth and proliferation rate than two previous days but better than positive control. In unmodified substrate (10 min collecting time), growth rate is still increasing unlike other modified and unmodified nano-fibrous substrates (40 min collecting time). In both modified and unmodified nano-fibrous substrates with 70 min collecting time, the growth rate reduces in comparison to two previous days. However, in all defined nano-fiber substrates, the rate of growth and proliferation are significantly better than the control. After 14 days, higher growth and proliferation rate was only seen on modified substrate with more roughness (70 min collecting time), compared to other substrates and positive control. This point confirms that the longer time frame for collecting electrospinning nano-fiber substrate leads to the creation of a proper structure similar to ECM. Based on the results achieved, it can be said that surface modification of PCL nano-fiber substrate by O_2_ plasma can improve hydrophilicity and initial cellular attachment, and finally increases the rate of growth and proliferation of HDFs (Fig. 3a). For OSTs, the substrate with 70 min collecting time shows progressive rate of growth and proliferation until 14 days compared to other substrates and control as shown in Fig. 3b. After 14 days on the substrate with 70 min collecting time, the rate of cellular proliferation shows a slower process. When these results are compared with other substrates, including the control, the substrate with 70 min collecting time displays the highest level of cellular proliferation in 14 and 18 days of cell culture. According to these results, the substrate with 70 min collecting time, without surface modification, improves in hydrophilicity and shows excellent condition for supporting OSTs compared to HDFs at 18 days of cell culture.

### HDFs and OSTs morphology on PCL substrates

The morphology evaluation of the cultured cells on the surface of substrates was performed by SEM after 48 h cell culturing (Fig. 4). As it is evident in the images, cells have suitable connection with the surface of substrates which confirms the results of the MTT assay. Nano-fibers are gradually covered by highest number of cells because they provide ECM natural-like environment for HDFs and OSTs. In tissue engineering, designing and production of the substrates are considered as important tasks because substrates play the same role as ECM in the body. The results of this study show that those substrates with nano-topographical feature have a significant impact on the cells, and thus on the success of engineered tissue. Finally, it can be concluded that cell fate depends on a combination of different important elements juxtaposing with each other (Irani et al. 2013; Adams 2002).

### Alizarin red staining and calcium content assay

Calcification of substrate is a first characteristic and standard marker for osteogenic differentiation process (Knezevic et al. 2010; Domingos et al. 2013). In this condition, using Alizarin red stain, the free calcium ions present in the substrate form sediments. Images from alizarin red staining show a substrate with 70 min collecting time has maximum sedimentation (Fig. 5). In addition, intracellular calcium content of OSTs is an indication of Ca content test. As shown in Fig. 6, the substrate with 70 min collecting time resulted in highest calcification of OSTs substrates. It is noteworthy that the rate of mineralization of OSTs cultured on the substrate is reduced after 10 days. Indeed, for providing essential Ca ions in osteogenesis process the cells must replace H with Ca, therefore, in extracellular environment presence of H results in acidification of the system under static condition. Those conditions can reduce rate of cell proliferation. Despite study on osteoblast there is a pH increase up to 7.8 in extracellular which can induce collagen synthesis and alkaline phosphatase activity (Dehghan et al. 2014). According to these results, all designed substrates, especially the 70 min collecting time could support OSTs osteogenic differentiation under appropriate conditions.

## Conclusion

One key challenge in guided tissue formation is to design and fabricate substrates with desirable surface properties, convenient for cell adhesion and proliferation that can regulate the process of tissue engineering. In this study, HDFs behavior was studied on modified and unmodified of PCL-based nano-fibrous mat spinning at three different collecting times. It was observed that HDFs showed higher cell adhesion strength on O_2_ plasma modified PCL substrates than on unmodified and positive control, while OSTs on unmodified PCL nano-fibers showed special cellular attachment on the first day. After that the comparison of growth and proliferation between OSTs and HDFs showed OSTs of higher rate than HDFs. In summary, based on the results obtained in this study it can be suggested that a substrate can be designed more or less similar structure of ECM of human body. It is noteworthy that the substrate with high collecting time (70 min) was suitable and desirable topography for OSTs. At the end, plasma PCL nano-fibrous electrospun mats with 70 min collecting time can be used as a substrate for bone tissue engineering.
